# Short-term knowledge gains and learner satisfaction after a basic radiology course for family medicine residents in a 3-dimensional virtual environment: a multi-cohort pretest–posttest study in Spain

**DOI:** 10.3352/jeehp.2026.23.25

**Published:** 2026-08-07

**Authors:** Alberto Pino-Postigo, Rocio Lorenzo-Alvarez, Dolores Dominguez-Pinos, Eugenio Lázaro Navarro-Sanchis, Eduardo Ochando-Pulido, Miguel Jose Ruiz-Gomez, Francisco Sendra-Portero

**Affiliations:** 1 Department of Radiology and Physical Medicine, Faculty of Medicine, University of Malaga, Malaga, Spain; 2 Department of Radiology, Hospital HLA El Angel, Malaga, Spain; 3 Department of Human Physiology, Faculty of Medicine, University of Malaga, Malaga, Spain; 4 Department of Emergency and Intensive Care, Hospital de la Axarquia, Velez-Malaga, Spain; 5 Department of Radiology, Hospital Universitario Virgen de la Victoria, Malaga, Spain; 6 IBIMA Plataforma BIONAND, Instituto de Investigación Biomédica de Málaga, Málaga, Spain; 7 Department of Radiology, Hospital Regional de Malaga, Malaga, Spain; Hallym University, Korea

**Keywords:** Family practice, Internship and residency, Radiology education, Computer simulation, Educational measurement, Spain

## Abstract

**Purpose:**

This study aimed to evaluate a structured basic radiology course for Family Medicine residents delivered in a 3-dimensional (3D) virtual environment (Second Life). We hypothesized that participation would be associated with higher post-course knowledge test scores and high learner satisfaction.

**Methods:**

A quasi-experimental multi-cohort pretest–posttest study was conducted across 3 consecutive cohorts of Family Medicine residents in Spain in 2019. Ninety-six participants engaged in a 15-day course combining synchronous and asynchronous activities. Sixty-five participants provided paired pre- and post-intervention knowledge assessments, which were analyzed using paired t-tests. Learner satisfaction was evaluated using a structured questionnaire with Likert-scale items, numerical ratings (0–10), and open-ended responses. Secondary exploratory comparisons between cohorts used the Kruskal–Wallis test for 0–10 rating scales. For Likert-scale items, pairwise comparisons between cohorts were performed using Mann–Whitney U tests, with Bonferroni correction. Qualitative data from open-ended responses were analyzed using thematic coding.

**Results:**

Participants had significantly higher post-course knowledge test scores, increasing from 47.4±11.6 to 58.2±11.9 (mean difference, 10.8; P<0.001; paired-sample Cohen’s d [dz]=0.67). High agreement was observed across most satisfaction items, with median scores ranging from 4 to 5. Content relevance, usefulness for clinical practice, and instructor performance received the highest ratings. Lower scores were observed for peer interaction and platform usability.

**Conclusion:**

A structured radiology course delivered in a 3D virtual environment was associated with higher immediate post-course knowledge test scores and high learner satisfaction among Family Medicine residents. The findings support the feasibility of repeated course delivery, although controlled studies are warranted.

## Graphical abstract

[Fig f6-jeehp-23-25]

## Introduction

### Background/rationale

Basic radiographic interpretation is an essential competency for family physicians in primary care and emergency settings [1]. However, radiology training during Family Medicine residency remains limited and heterogeneous, suggesting a gap between clinical demands and formal education [2].

Immersive 3-dimensional (3D) virtual environments offer interactive and flexible learning opportunities for medical education. Previous studies have shown that Second Life is a feasible platform for radiology education, with high learner acceptance and learning outcomes comparable to face-to-face teaching [3,4]. However, evidence regarding its use in Family Medicine residency remains limited. This study intervention incorporated experiential and case-based learning principles within an immersive collaborative environment.

### Objectives

This study evaluated knowledge test outcomes and learner satisfaction following a structured radiology course for Family Medicine residents delivered in a 3D virtual environment (Second Life) across 3 cohorts. We hypothesized that participation would be associated with higher immediate post-course knowledge test scores and high learner satisfaction.

## Methods

### Ethics statement

This study is a retrospective analysis of educational data collected during routine educational activities in 2019. Ethical approval for the analysis and publication of these data was granted by the Research Ethics Committee of the University of Málaga (CEUMA; approval no., 001-2023-H; January 25, 2023). Participants were assigned unique alphanumeric identifiers to link pre- and post-course assessments. Participation was voluntary. Participants were informed that de-identified data would be used for research and scientific dissemination, and questionnaire completion constituted informed consent.

### Study design

This quasi-experimental single-group pretest–posttest study was conducted across 3 independent cohorts of Family Medicine residents in January, September, and October 2019. All participants received the same intervention. Outcomes were evaluated using standardized multiple-choice tests administered before and after the course and a post-course satisfaction survey. The study was reported in accordance with the Transparent Reporting of Evaluations with Nonrandomized Designs (TREND) statement [5].

### Setting

**Fig. 1.**
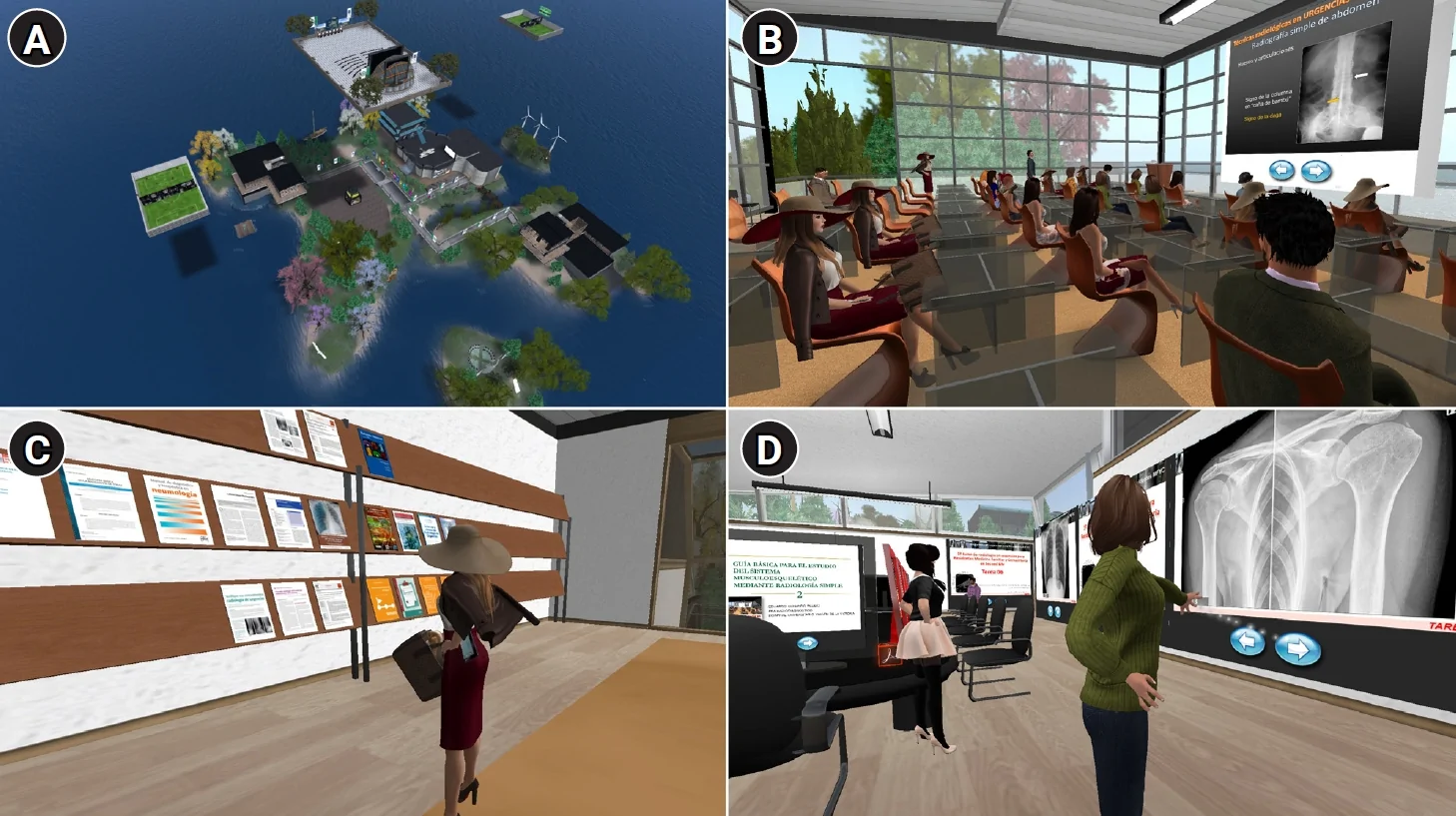
Screenshots of different scenarios in the virtual world Second Life used in this study. (A) Aerial view of the virtual island. (B) Classroom in the postgraduate building during a synchronous session. (C) Virtual library providing access to educational materials. (D) Hall displaying content from asynchronous tasks and recorded presentations.

The course took place on a virtual island in Second Life designed to simulate a university campus (Fig. 1A). Training was conducted in a postgraduate building containing areas for synchronous sessions (Fig. 1B), access to educational materials (Fig. 1C), and asynchronous activities, including recorded presentations (Fig. 1D). The course was delivered in 3 editions, allowing geographically distributed residents across Spain to participate remotely.

### Participants

The study population comprised Family Medicine residents in Spain recruited nationwide through social media announcements in collaboration with the Spanish Society of Family and Community Medicine (SEMFYC) and the Spanish Society of Medical Radiology (SERAM). Eligible participants were active residents who registered voluntarily. Enrollment was on a first-come, first-served basis, with a maximum of 40 participants per cohort and a €10 registration fee to encourage commitment and reduce dropout. Across the 3 editions, 115 residents enrolled and 96 participated (82 completed the course and 14 partially). Participants were recruited from multiple regions across Spain, and participation was not linked to formal academic evaluation or certification.

### Interventions

A training program titled *Radiology in Emergency Settings* was designed to develop Family Medicine residents’ skills in requesting and interpreting basic radiographs (Table 1, Supplement 1). The intervention was delivered in 3 editions. Each edition lasted 15 days and combined synchronous and asynchronous activities. Participants attended 3 live sessions (3.5 hours each), held 1 week apart and led by radiology faculty, with real-time interaction and discussion. The curriculum covered chest, abdominal, and musculoskeletal imaging, together with the appropriateness of imaging requests, using case-based learning. Between sessions, participants completed asynchronous activities focused on radiographic interpretation and imaging-based clinical decision-making.

### Outcomes

The primary outcome was the change in pre- and post-course knowledge test scores. Secondary outcomes were learner satisfaction and perceptions of the educational experience. Outcomes were also compared across the 3 cohorts.

### Data sources/measurement

Radiological knowledge was assessed using a 20-item multiple-choice test administered before and after the intervention, based on the SERAM question bank [6]. All questions addressed the interpretation of emergency plain radiographs covering the 3 course domains: chest, abdominal, and musculoskeletal radiology. The same test was used at both time points, with results withheld until completion of the post-intervention assessment. Scores were expressed as the percentage of correct responses. Test content and administration were identical across cohorts.

Learner satisfaction and perceptions of the educational experience were evaluated using a structured end-of-course questionnaire (Supplement 2) adapted from previously published instruments in virtual radiology education [7]. The questionnaire assessed prior familiarity with the platform, the learning environment, course content, instructor performance, and the interest and difficulty of asynchronous tasks using a 5-point Likert scale (1=strongly disagree to 5=strongly agree). Participants also rated selected course aspects on a 0–10 scale and provided open-ended comments. Test results and questionnaire responses are available in Dataset 1 and Dataset 2, respectively. Variable definitions are provided in Supplement 3. Internal consistency of the Likert-scale questionnaire was assessed using Cronbach’s alpha.

### Bias

Several potential sources of bias were considered. Voluntary participation may have introduced self-selection bias by favoring more motivated residents. The absence of a control group and randomization limits causal inference. To reduce procedural variability, the same course structure, content, and assessments were used across all cohorts.

### Study size

No a priori sample size calculation was performed. Sample size was pragmatically determined by course capacity (40 residents per cohort), a limit established to avoid performance problems associated with large numbers of concurrent avatars in the virtual environment. Across the 3 editions, 115 residents enrolled and 96 participated. The study was designed to assess within-group changes in radiological knowledge.

### Assignment method

The unit of assignment was the individual participant. All participants received the same intervention.

### Blinding (masking)

Blinding was not applicable, as all participants received the same intervention. Outcome assessment was based on objective test scores and de-identified questionnaires, reducing the risk of assessment bias.

### Unit of analysis

The unit of analysis was the individual participant, with outcomes including pretest–posttest differences in knowledge scores and questionnaire responses. As this matched the unit of assignment, no adjustment for clustering or hierarchical structure was required.

### Statistical methods

Pre- and post-course knowledge scores were compared using paired Student t-tests, with mean differences, 95% confidence intervals (CIs), and paired-sample Cohen’s d (dz) reported. Survey data were summarized descriptively. Secondary exploratory comparisons between cohorts used the Kruskal–Wallis test for 0–10 rating scales. For Likert-scale items, pairwise comparisons between cohorts were performed using Mann–Whitney U tests, with Bonferroni correction for the 3 pairwise comparisons. As the primary objective was to evaluate within-participant pre–post changes, no formal cohort-by-time interaction analysis was performed. The 0–10 scales are presented as median (Q1–Q3), and Likert-scale responses as percentages of agreement (4–5), neutral (3), and disagreement (1–2), with median (interquartile range [IQR]). Analyses were performed using R ver. 4.5.3 (The R Foundation for Statistical Computing, Vienna, Austria), with a significance level of P<0.05. Missing data were handled using available-case analysis without imputation. Qualitative data from open-ended responses were analyzed using inductive thematic coding. Responses were reviewed independently by the researchers, and themes and subcodes were defined through consensus.

## Results

### Participants

**Fig. 2.**
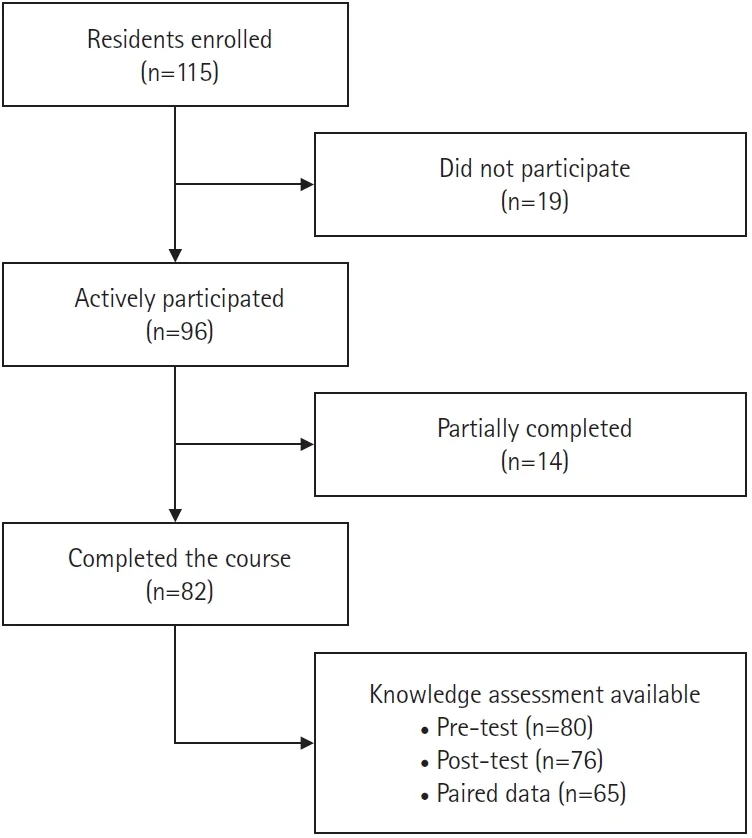
Flow diagram of participant recruitment, participation, and course completion across the study.

Fig. 2 shows the flow of recruitment, participation, course completion, and analysis. Of the 115 residents enrolled across the 3 editions, 96 participated in the course (82 completed and 14 partially). Pre- and post-course knowledge test data were available for 80 and 76 participants, respectively, with 65 paired datasets included in the primary analysis. Eighty-two participants completed the post-course satisfaction questionnaire. Among the 96 participants, 82 (85.4%) were women and 14 (14.6%) were men. The cohort included 50 first-year (R1, 52.1%), 24 second-year (R2, 25.0%), 15 third-year (R3, 15.6%), and 7 fourth-year (R4, 7.3%) residents. Demographic characteristics by cohort are presented in Table 2. Participants were recruited from 25 hospitals in 23 cities across 10 Spanish autonomous communities. No major deviations from the planned intervention or assessment procedures occurred across cohorts.

### Main results

**Fig. 3.**
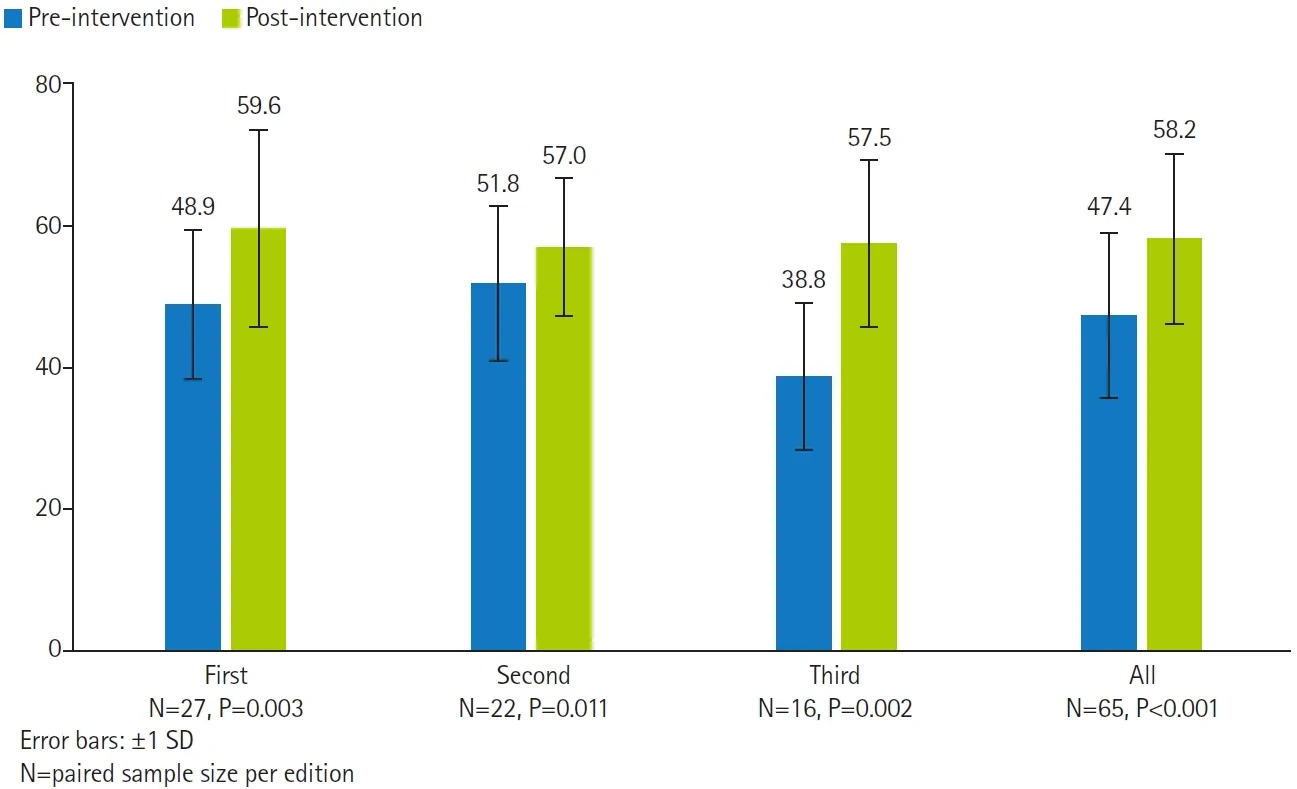
Pre- and post-intervention test scores. Bars represent mean scores. P-values were obtained using paired Student t-tests. SD, standard deviation.

Participants with paired pre- and post-course assessments (n=65) showed significant improvement in test scores after the course (Fig. 3, Supplement 4), increasing from 47.4±11.6 to 58.2±11.9 (mean difference, 10.8; 95% CI, 6.8–14.9; P<0.001; paired-sample Cohen’s d [dz]=0.67; 95% CI, 0.40–0.93). Significant increases were observed in all 3 cohorts. Baseline characteristics did not differ significantly between participants with complete paired assessments and those with pre-course assessment only, including baseline knowledge scores (P=0.482), sex (P=0.745), and residency year (P=0.556) (Supplement 5).

**Fig. 4.**
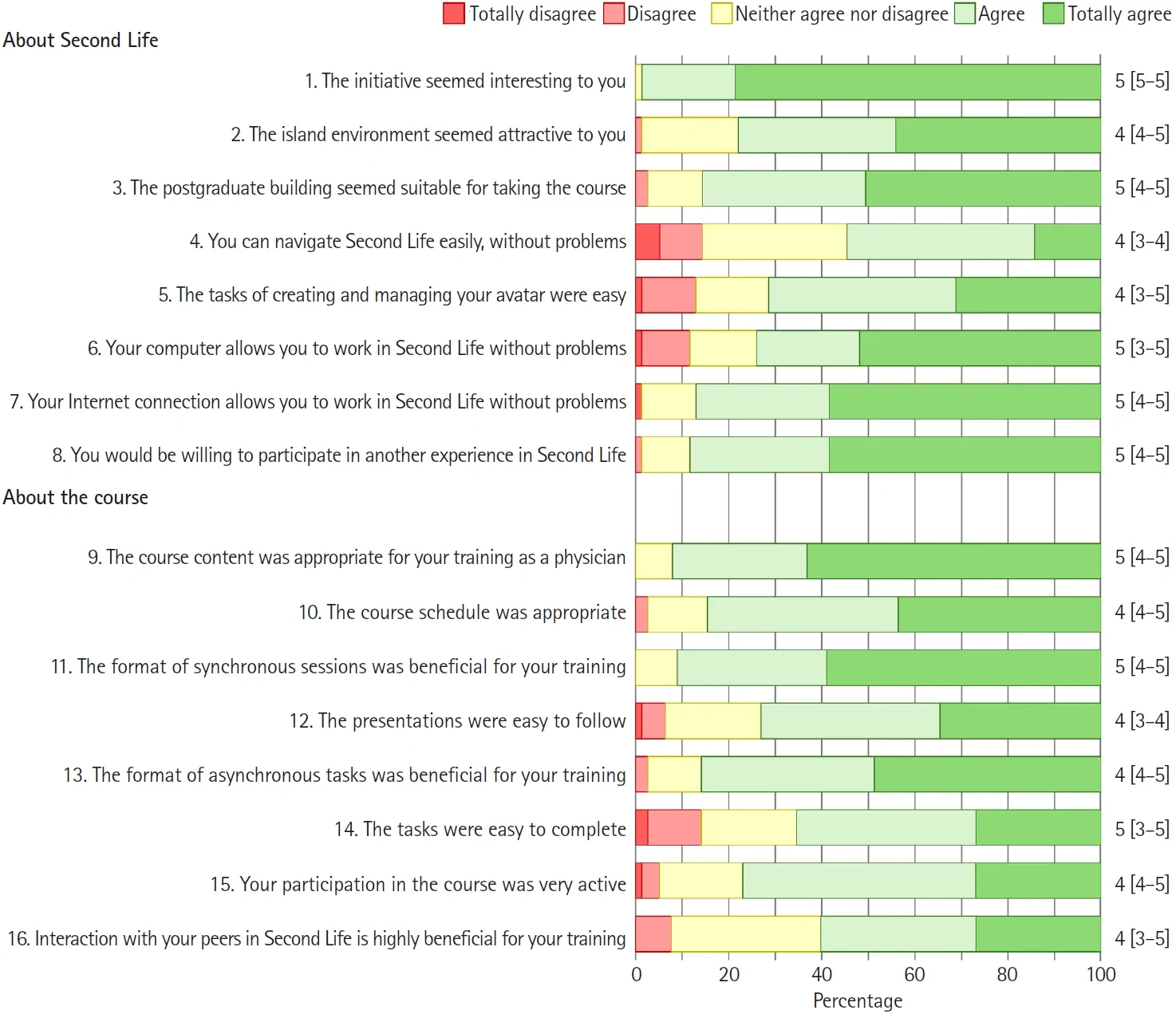
Bar charts showing responses to questions About Second Life and the course. Data are presented as percentages of responses for each Likert category (1–5). Values displayed correspond to median [interquartile range].

**Fig. 5.**
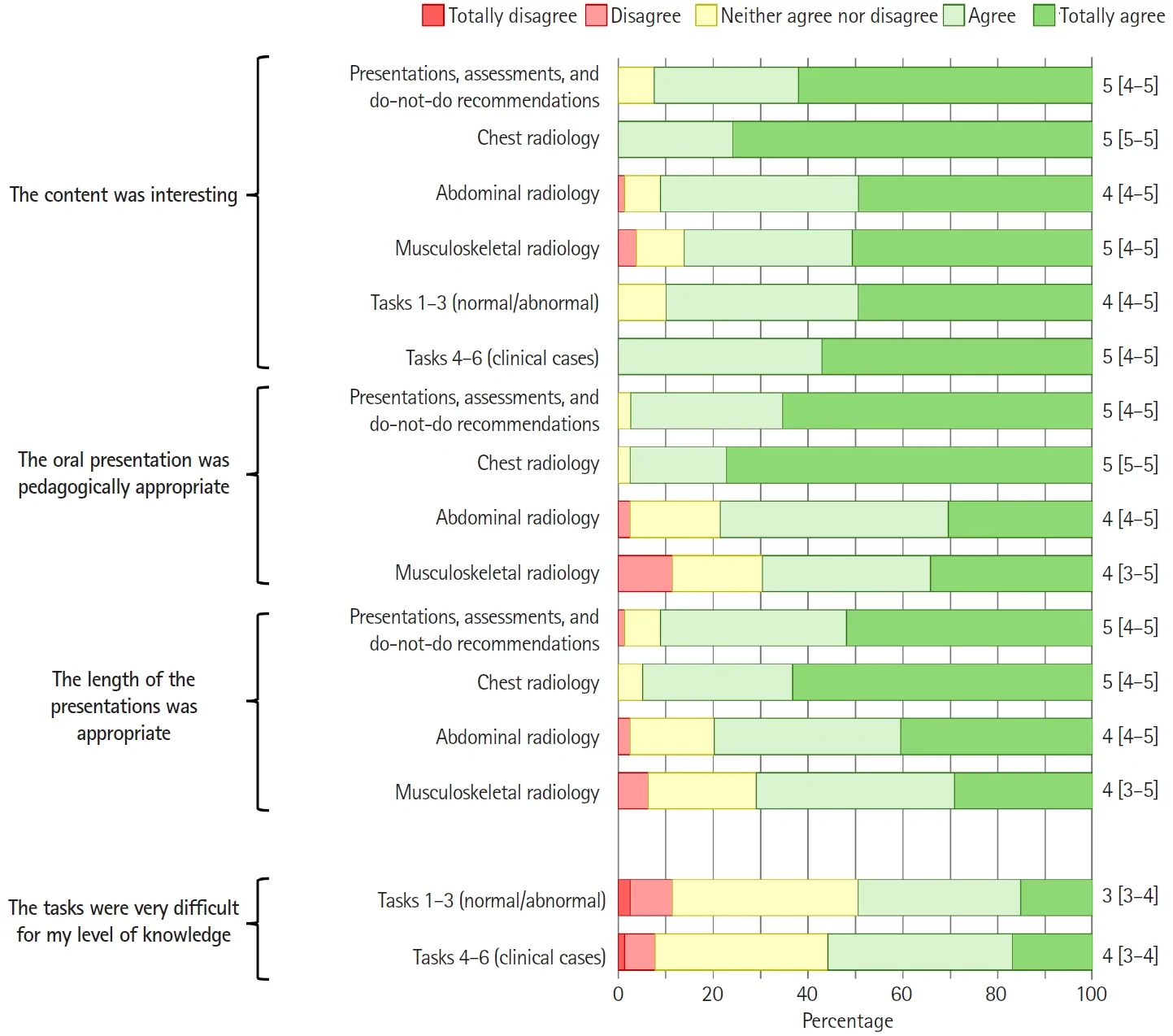
Bar charts showing responses to questions about course content, presentations, and tasks. Data are presented as percentages of responses for each Likert category (1–5). Values correspond to median [interquartile range].

Eighty-two participants completed the post-course questionnaire (32, 30, and 20 in the first, second, and third editions, respectively). The overall questionnaire yielded a Cronbach’s alpha of 0.93. Most Likert-scale items showed high agreement (Figs. 4, 5). The initiative, educational content, synchronous sessions, willingness to participate again, and the chest radiology module received the highest ratings (median, 4–5 [IQR, 4–5]; chest module: median, 5 [IQR, 5–5]). Lower ratings were observed for ease of navigation, avatar management, peer interaction, and task difficulty (median, 3–4 [IQR, 3–5]). After Bonferroni correction, only “The presentations were easy to follow” differed significantly between Editions 1 and 2 (median, 5 [IQR, 4–5] vs. median, 4 [IQR, 3–4]; adjusted P=0.037). These between-cohort comparisons should be interpreted as exploratory.

Participants reported high 0–10 ratings across all aspects of the course (Table 3), with median scores ranging from 7 to 9. Educational content, usefulness for training, and instructors received the highest ratings (median=9), whereas interaction with peers and connectivity to Second Life received comparatively lower ratings. Most variables showed no significant differences between course editions (cohorts). However, significant between-cohort differences were observed for the island environment, interaction with peers, and connectivity to Second Life.

Open-ended responses (Supplement 6) were grouped into advantages, disadvantages, and suggestions (Supplement 7). Participants most frequently highlighted the practical usefulness and clinical relevance of the course (n=19), describing it as “a very creative, useful, and comfortable activity” (E1-01). The accessibility of the virtual format and remote participation were also positively valued (n=7). The main disadvantages were technical and usability issues (n=8), including connectivity problems and avatar management, with one participant noting: “It took time to learn how to handle the avatar properly” (E2-26). Session length and cognitive fatigue were also commonly mentioned (n=6). Suggestions focused on shorter sessions distributed over more days (n=6) and access to recorded sessions and additional support materials (n=3–4).

## Discussion

### Key results

Participation in the course was associated with higher post-course knowledge test scores and high learner satisfaction. Participants reported positive perceptions of the virtual environment, educational content, and teaching methodology, particularly the relevance of the course content, instructor performance, and synchronous sessions. Open-ended responses reinforced these findings while identifying technical issues and session length as the main limitations.

### Interpretation

These findings indicate that participation in a structured radiology course delivered in a 3D virtual environment was associated with higher post-course knowledge test scores among Family Medicine residents. High ratings for content relevance and clinical usefulness suggest that participants considered the course educationally valuable. This is consistent with previous reports describing limited competency in chest radiograph interpretation in emergency settings [8]. The chest radiology module received the highest ratings among the course topics. Variability in task difficulty, peer interaction, and navigation highlights challenges in implementing immersive learning platforms. Lower satisfaction scores in later editions, particularly for connectivity and interaction, may reflect differences in user experience or cohort characteristics. These findings underscore the importance of platform usability and technical performance for learner experience in 3D virtual environments [9,10].

### Comparison with previous studies

These findings are consistent with prior research on radiology education in virtual environments, where learning outcomes in immersive platforms are comparable to face-to-face settings in both interpretive workshops [4] and problem-based learning [11]. Together with reports of high learner satisfaction [3], they support immersive learning as a useful strategy with potential applications in postgraduate training. The technical and usability challenges identified in the present study are also consistent with previous reports [3,4,11]. Recent studies using immersive virtual reality head-mounted devices have reported increased motivation and perceived usefulness in undergraduate radiology education, attributed to improved spatial understanding [12], although evidence remains limited, particularly in diagnostic radiology. Despite the use of a screen-based environment, our results showed high engagement and perceived usefulness, supporting the view that effectiveness depends not only on immersion but also on usability and system design [13]. These findings suggest that instructional design and clinical relevance may be as important as immersion.

### Limitations

This study has several limitations. The quasi-experimental pretest–posttest design without a control group limits causal inference, as factors other than the intervention may have contributed to the higher post-course knowledge test scores. Voluntary participation may have introduced self-selection bias. Although paired assessments were available for only 65 participants, baseline characteristics were similar between participants with paired and pre-course-only assessments; however, attrition bias cannot be excluded. The use of the same 20-item knowledge test before and after the course may have introduced practice effects, and future studies should consider parallel test forms or delayed post-course assessments. Differences in residency year and previous training may also have influenced outcomes. Furthermore, the study assessed only short-term knowledge test performance and self-reported satisfaction, without evaluating long-term retention, clinical impact, or diagnostic performance. Whether these findings persist over time remains unknown. Future studies should assess long-term retention and transfer to clinical practice. Finally, the use of a single virtual platform (Second Life) may limit generalizability to other immersive learning environments.

### Generalizability

Findings may be generalizable to similar educational contexts involving healthcare professionals. Although participants were recruited from multiple regions across Spain, voluntary first-come, first-served enrollment limits population representativeness and generalizability. The successful implementation of the course across 3 independent cohorts, together with favorable learner evaluations, supports the feasibility and consistent delivery of this educational approach in routine educational settings.

### Suggestions

Future research should evaluate the long-term effects of this intervention on knowledge retention, clinical decision-making, and diagnostic performance, preferably using controlled comparative designs. Improving platform usability and technical performance may enhance learner experience, particularly in large synchronous sessions. An extended version of the course for radiology residents is currently under evaluation. Further studies should also explore adaptation of this model to other specialties and educational levels.

### Conclusion

A structured radiology course delivered in a 3D virtual environment was associated with higher immediate post-course knowledge test scores among Family Medicine residents and high learner satisfaction. The successful implementation of the course across 3 independent cohorts supports the feasibility of repeated course delivery in routine educational settings. Participants also gave favorable evaluations of the clinically relevant, case-based content. Further controlled studies using validated parallel assessments and delayed follow-up are warranted to evaluate long-term knowledge retention and transfer to clinical performance.

## Figures and Tables

**Figure f6-jeehp-23-25:**
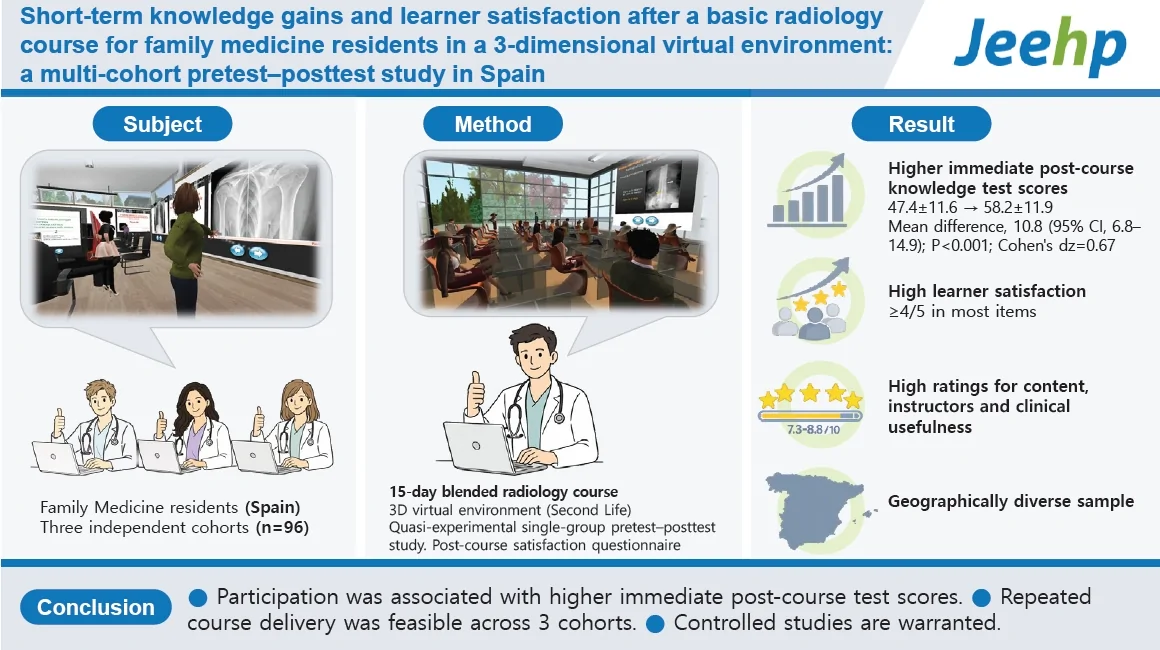


**Table 1.** Overview of the course schedule and content

Day	Hour	Clinical presentation
Wednesday 1	17:00	Course introduction and initial assessment (Pre-intervention test).
	17:30	Systematic approach to reading chest X-rays.
	18:30	Imaging techniques in abdominal emergencies.
	19:30	Musculoskeletal radiography: Upper extremity.
Asynchronous tasks		
Wednesday 2	17:00	Do-not-do recommendations in emergency radiology.
	17:30	Chest case studies I.
	18:30	Radiological management of the acute abdomen.
	19:30	Musculoskeletal radiography: Lower extremity.
Asynchronous tasks		
Wednesday 3	17:00	Chest case studies II.
	18:00	Abdominal case studies.
	19:00	Musculoskeletal radiography: Spine.
	20:00	Course closing and final assessment (Post-intervention test).

**Table 2.** Demographic characteristics of participants by cohort

Edition	R1	R2	R3	R4	Men	Women
First (36/32)	23/21	6/6	5/4	2/1	4/4	32/28
Second (34/30)	15/14	14/11	3/3	2/2	5/5	29/25
Third (26/20)	12/11	4/3	7/4	3/2	5/3	21/17
All (96/82)	50/46	24/20	15/11	7/5	14/12	82/70

Values are presented as residents participating/completing the course.

**Table 3.** Participants’ global ratings of key project aspects (0–10 scale)

Project aspect	First edition (Cohort 1) (n=32)	Second edition (Cohort 2) (n=30)	Third edition (Cohort 3) (n=20)	All editions (n=82)	P-value
Overall experience	9 (8–10)	8 (8–9)	8 (8–9)	8.5 (8–10)	0.386
Project organization	9 (8–10)	9 (8–10)	9 (8–9.5)	9 (8–10)	0.435
The island environment	9 (8–10)	8 (7–10)	7.5 (6–9)	8 (7–10)	0.025^*^
Educational content	9 (8–10)	9 (8–10)	9 (7.5–9)	9 (8–10)	0.222
Usefulness for your training	9 (8–10)	9 (8–10)	9 (8–9)	9 (8–10)	0.410
Instructors	9 (8–10)	9 (8–10)	9 (8–9)	9 (8–10)	0.245
Interaction with peers	8 (7–9)	7 (5–8)	7 (5.5–8.5)	8 (6–9)	0.018^*^
Synchronous sessions	9 (8–10)	8 (8–9)	8 (7–9)	8 (8–9)	0.060
Asynchronous tasks	8 (7–10)	8 (7–9)	8 (6–9)	8 (7–9)	0.140
Connectivity to Second Life	9 (8–10)	7.5 (6–9)	7 (5–8)	8 (6–9)	<0.001^*^

Values are presented as median (Q1–Q3). P-values were obtained using the Kruskal-Wallis test to compare the 3 course editions (cohorts).

* P<0.05 (statistically significant differences).

## Footnotes

**Data availability**
Data files are available from https://doi.org/10.7910/DVN/8SPGWU Dataset 1. Raw data of the pre- and post-intervention test results.

Dataset 2. Raw data of the questionnaire.

## Supplementary materials

Supplement 1. Detailed description of the virtual course program translated into English.

Supplement 2. Questionnaire used for course evaluation translated into English.

Supplement 3. Variable dictionary for datasets 1 and 2.

Supplement 4. Results of the multiple-choice tests conducted at the beginning and at the end of the course.

Supplement 5. Comparison of baseline characteristics between participants with complete paired assessments and those with pre-course assessment only.

Supplement 6. List of open-ended comments included in the survey translated into English.

Supplement 7. Key subcodes for first-level codes (advantages, disadvantages, and suggestions) in open-ended residents’ feedback.

Supplement 8. Audio recording of the abstract.
