# Supplementary material for: Short-term knowledge gains and learner satisfaction after a basic radiology course for family medicine residents in a 3-dimensional virtual environment: a multi-cohort pretest–posttest study in Spain
Source: J Educ Eval Health Prof. 2026 Aug 7;23:25. doi: 10.3352/jeehp.2026.23.25 (PMC13575735; doi:10.3352/jeehp.2026.23.25)
Supplement: Supplementary file 1 [file jeehp-23-25-suppl1.pdf]

Objectives

To improve competencies in the interpretation of radiographs in emergency medical settings.

Specific Objectives

1. *To develop learning of interpretative skills for reading radiographs in emergency settings.*
2. *To improve the appropriateness of radiograph requests in emergency care.*
3. *To identify the indications and "do-not-do" recommendations for radiographs in emergency medical settings.*
4. *To integrate all of the above in a participatory environment with a strong emphasis on voice chat.*

Requirements

1. *Be a Family Medicine resident.*
2. *Create an account in Second Life.*
3. *Download the Second Life Viewer.*
4. *Have a microphone and headphones.*
5. *Check in advance, before the course, that there are no technical issues.*

Professors

María Dolores Dominguez-Pinos[1]
Eugenio L. Navarro-Sanchis[2]
Eduardo Ochando-Pulido[3]
Francisco Sendra-Portero[4]

1. ***Specialist Physician in Radiology and in Family and Community Medicine. Staff Radiologist, Hospital Universitario Virgen de la Victoria.***
2. ***Specialist Physician in Radiology. Associate Professor, University of Málaga. Staff Radiologist, Hospital Regional Universitario de Málaga.***
3. ***Specialist Physician in Radiology. Staff Radiologist, Hospital Universitario Virgen de la Victoria.***
4. ***Specialist Physician in Radiology. Full Professor, University of Málaga.***

Integrated into the educational innovation project of the University of Málaga PIE17-113: "Deepening the Learning of Radiology in 3D Immersive Environments."

Resident Training

Interpretive Skills

Organized by

Laboratorio de Radiología Digital y Educación Electrónica. Grupo TIC128 del PAIDI*

Asociación de Radiólogos del Sur

Departamento de Radiología y Medicina Física, Oftalmología y Otorrinolaringología - UMA

With the scientific endorsement of

seram
Sociedad Española de Radiología Médica

A certificate of participation for 16 hours will be issued.

Intended for: Family Medicine Resident Physicians.

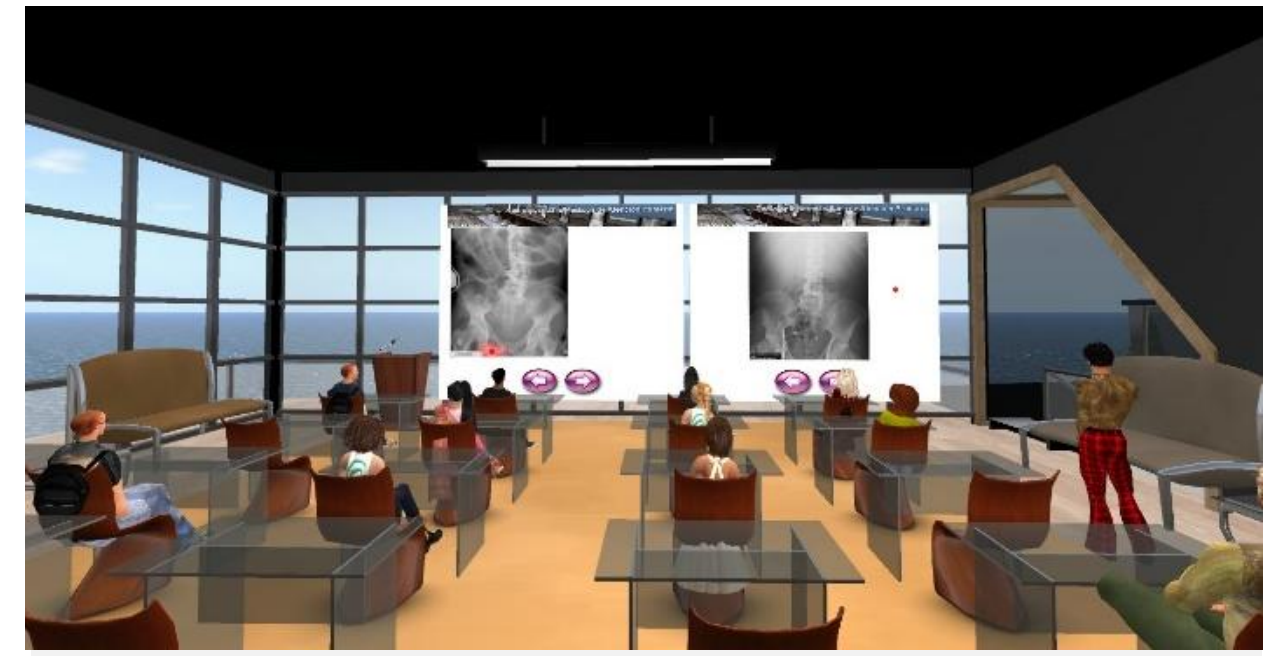

1st Emergency Radiology Course for Family Medicine Residents in Second Life

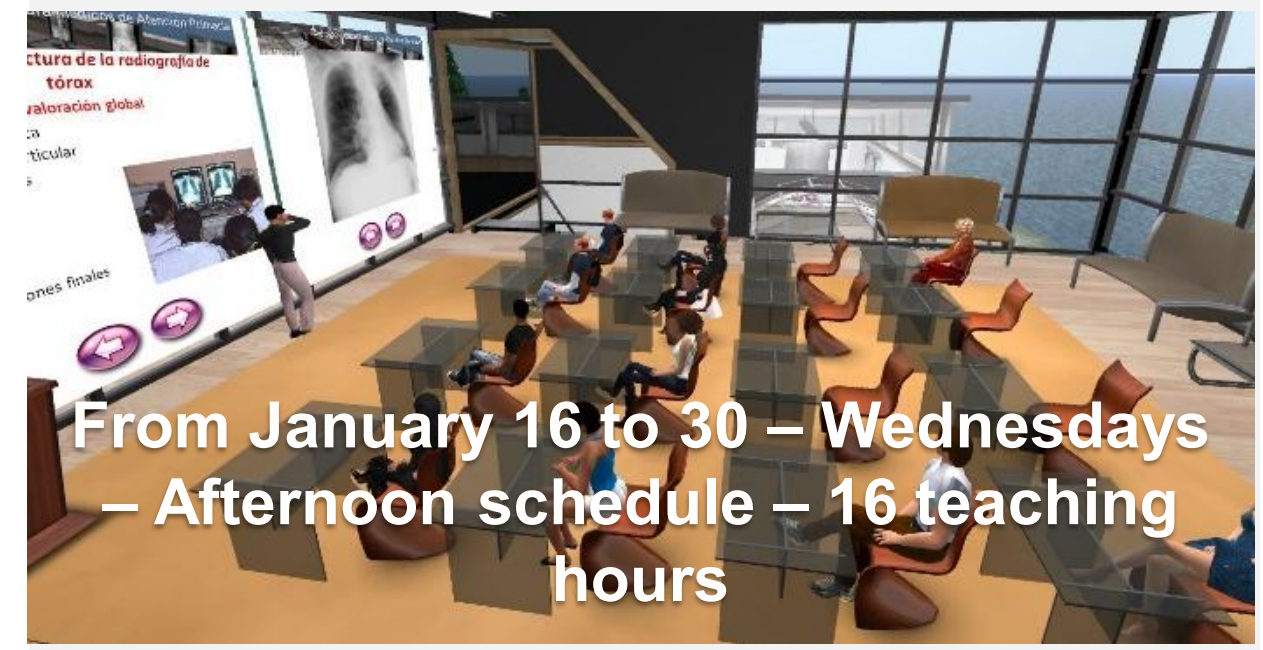

Second Life – The Medical Master Island – Postgraduate Building

http://maps.secondlife.com/secondlife/Medical%20Master%20Island/119/90/22

How to Lose Your Fear of X-rays in Emergency Care!

PROGRAM

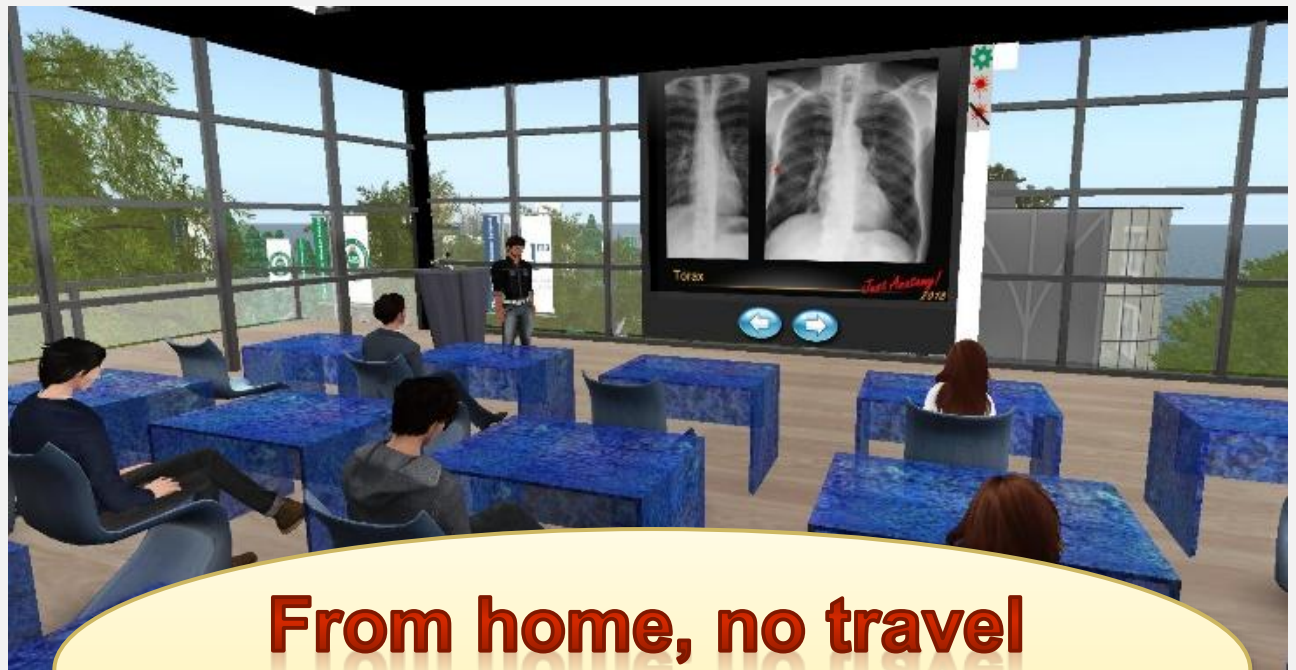

From home, no travel required!

Second Life

All relevant information about Second Life is available on its website: www.secondlife.com

Second Life is a user-created community that can be used for a variety of purposes, including educational ones (which is precisely our aim).

To participate in this project, it is essential to register in Second Life and create an avatar.

Online course in the immersive environment of Second Life

In-person learning beyond the classroom!

Emergency Radiology Course

The course is exclusively aimed at Family Medicine residents.

It will consist of three face-to-face sessions, each lasting 3.5 hours.

Sessions will take place from 17:00 to 20:30.

Between sessions, practical cases will be assigned, which enrolled participants must complete before the next session, for a total equivalent of 6 hours.

Emphasis will be placed on systematic reading, the resolution of clinical cases, and the appropriate indication of imaging tests.

There will be an initial and a final assessment in order to evaluate progress in the knowledge acquired.

Case-solving, a true meeting point!

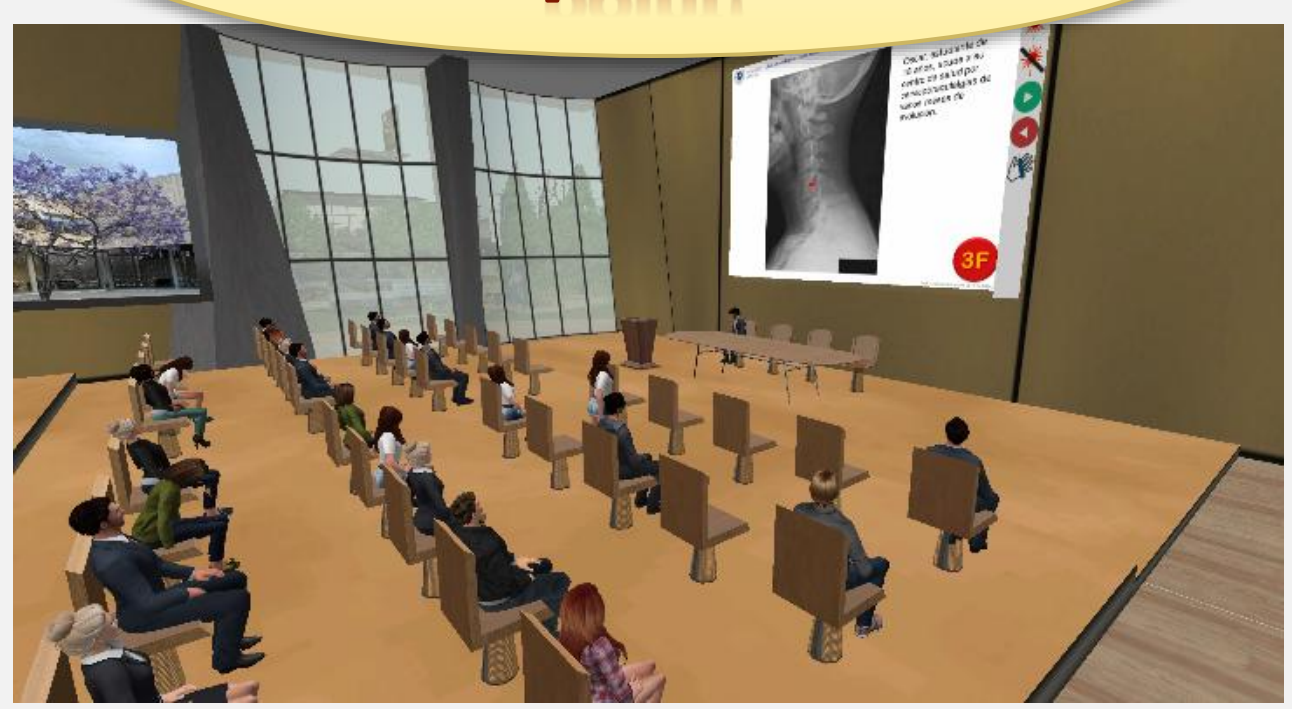

PROGRAM

Schedule

Course Content	Date
Course Introduction. Initial Assessment.	16 Jan
Systematic Approach to Chest X-ray Interpretation	16 Jan
Radiological Techniques in Abdominal Emergencies.	16 Jan
Musculoskeletal Radiography – Upper Limb.	16 Jan
"Do-not-do" Recommendations in Emergency Radiology.	23 Jan
Chest Clinical Cases I.	23 Jan
Radiological Management of the Acute Abdomen.	23 Jan
Musculoskeletal Radiography – Lower Limb	23 Jan
Chest Clinical Cases II.	30 Jan
Abdominal Clinical Cases.	30 Jan
Musculoskeletal Radiography – Spine.	30 Jan
Course Closing. Final Assessment	30 Jan

REGISTRATION: 10 €

Limited to 40 participants (on a first-come, first-served basis).

Please make a bank transfer in the name of ***ASOCIACIÓN DE RADIÓLOGOS DEL SUR*** *to the following account:*

ESXX XXXX XXXX XXXX XXXX

Please include the reference: ***SECOND LIFE COURSE – FAMILY MEDICINE RESIDENTS***

Send proof of payment by email to **cursos@radiologiabasica.org** *indicating:*

- *Full name*
- *Hospital, city, and year of residency*
