# Supplementary material for: Short-term knowledge gains and learner satisfaction after a basic radiology course for family medicine residents in a 3-dimensional virtual environment: a multi-cohort pretest–posttest study in Spain
Source: J Educ Eval Health Prof. 2026 Aug 7;23:25. doi: 10.3352/jeehp.2026.23.25 (PMC13575735; doi:10.3352/jeehp.2026.23.25)
Supplement: Supplementary file 2 [file jeehp-23-25-suppl2.docx]

**QUESTIONNAIRE**

***About Second Life***

Did you know about Second Life before this experience (Y/N)

*If you answer is affirmative, please indicate the circumstances in which you learned about Second Life*

*Please complete the following questions within the box, rating from 1 to 5*

*(1 totally disagree, 2: disagree, 3: neither agree nor disagree, 4: agree, 5: totally agree)*

The initiative seemed interesting to you

The island environment seemed attractive to you

The postgraduate building seemed suitable for taking the course

You can navigate Second Life easily, without problems

The tasks of creating and managing your avatar were easy

Your computer allows you to work in Second Life without problems

Your Internet connection allows you to work in Second Life without problems

You would be willing to participate in another experience in Second Life

***About the course***

The course content was appropriate for your training was as a physician

The course schedule was appropriate

The format of synchronous sessions was beneficial for your training

The presentations were easy to follow

The format of asynchronous tasks was beneficial for your training

The tasks were easy to complete

Your participation in the course was very active

Interaction with your peers in Second Life is highly beneficial for your training

**QUESTIONNAIRE**

*Please complete the following questions within the box, rating from 1 to 5*

*(1 totally disagree, 2: disagree, 3: neither agree nor disagree, 4: agree, 5: totally agree)*

**Presentations, assessments, and not-to-do recommendations.**

The contents were interesting

The oral presentation was pedagogically appropriate

The quality of the displayed presentations was good

The length of the presentations was appropriate

**Thoracic Radiology.**

The contents were interesting

The oral presentation was pedagogically appropriate

The quality of the displayed presentations was good

The length of the presentations was appropriate

**Abdominal radiology.**

The contents were interesting

The oral presentation was pedagogically appropriate

The quality of the displayed presentations was good

The length of the presentations was appropriate

**Musculoskeletal Radiology.**

The contents were interesting

The oral presentation was pedagogically appropriate

The quality of the displayed presentations was good

The length of the presentations was appropriate

**Tasks 1, 2, and 3 (normal - abnormal)**

The contents were interesting

The tasks were very difficult for my level of knowledge

**Tasks 4, 5, and 6 (clinical cases)**

The contents were interesting

The tasks were very difficult for my level of knowledge

**QUESTIONNAIRE**

***Important! 0 to 10 rating***

*Please complete the following questions within the box, rating from 1 to 10*

Overall experience

Project organization

The Island environment

Educational content

Usefulness for your training

Instructors

Interaction with peers

Synchronous sessions

Asynchronous tasks

Connectivity to Second Life

***Open comments***

*If you wish to add anything, please use the box below*
