# Supplementary material for: Short-term knowledge gains and learner satisfaction after a basic radiology course for family medicine residents in a 3-dimensional virtual environment: a multi-cohort pretest–posttest study in Spain
Source: J Educ Eval Health Prof. 2026 Aug 7;23:25. doi: 10.3352/jeehp.2026.23.25 (PMC13575735; doi:10.3352/jeehp.2026.23.25)
Supplement: Supplementary file 3 [file jeehp-23-25-suppl3.docx]

**Supplement 3.** Variable dictionary for datasets 1 and 2

| **Variable** | **Description** |
| --- | --- |
| **Dataset1** |  |
| Participant_ID | Anonymous participant code |
| Sex | Participant sex |
| Residency_year | Residency yeaR |
| Pre_score | Percentage score in pre-intervention test |
| Post_score | Percentage score in post-intervention test |
| Test_status | Availability of knowledge test data for each participant. Used to identify participants included in paired and unpaired analyses. |
| **Dataset2** | **About Second Life** |
| Cohort | Course edition (1–3) |
| Q1 | The initiative seemed interesting to you |
| Q2 | The island environment seemed attractive to you |
| Q3 | The postgraduate building seemed suitable for taking the course |
| Q4 | You can navigate Second Life easily, without problems |
| Q5 | The tasks of creating and managing your avatar were easy |
| Q6 | Your computer allows you to work in Second Life without problems |
| Q7 | Your Internet connection meets the requirements to work in Second Life without problems |
| Q8 | You would be willing to participate in another experience in Second Life |
| **Dataset2** | **About the course** |
| Q9 | The course content was appropriate for your training was appropriate for your training as a physician |
| Q10 | The course schedule was appropriate |
| Q11 | The format of synchronous sessions was beneficial for your training |
| Q12 | The presentations were easy to follow |
| Q13 | The format of asynchronous tasks was beneficial for your training |
| Q14 | The tasks were easy to complete |
| Q15 | Your participation in the course was very active |
| Q16 | Interaction with your peers in Second Life is highly beneficial for your training |
| **Dataset2** | **Presentations, assessments, and not-to-do recommendations.** |
| Q17 | The contents were interesting |
| Q18 | The oral presentation was pedagogically appropriate |
| Q19 | The quality of the displayed presentations was good |
| Q20 | The length of the presentations was appropriate |
| **Dataset2** | **Thoracic Radiology.** |
| Q21 | The contents were interesting |
| Q22 | The oral presentation was pedagogically appropriate |
| Q23 | The quality of the displayed presentations was good |
| Q24 | The length of the presentations was appropriate |
| **Dataset2** | **Abdominal radiology.** |
| Q25 | The contents were interesting |
| Q26 | The oral presentation was pedagogically appropriate |
| Q27 | The quality of the displayed presentations was good |
| Q28 | The length of the presentations was appropriate |
| **Dataset2** | **Musculoskeletal Radiology.** |
| Q29 | The contents were interesting |
| Q30 | The oral presentation was pedagogically appropriate |
| Q31 | The quality of the displayed presentations was good |
| Q32 | The length of the presentations was appropriate |
| **Dataset2** | **Tasks 1, 2, and 3 (normal - abnormal)** |
| Q33 | The contents were interesting |
| Q34 | The tasks were very difficult for my level of knowledge |
| **Dataset2** | **Tasks 4, 5, and 6 (clinical cases)** |
| Q35 | The contents were interesting |
| Q36 | The tasks were very difficult for my level of knowledge |
| **Dataset2** | **Student global ratings of key project aspects (0–10 Scale)** |
| Experience | Overall experience |
| Organization | Project organization |
| Environment | The island environment |
| Content | Educational content |
| Usefulness | Usefulness for your training |
| Instructors | Instructors |
| Peer_interaction | Interaction with peers |
| Sessions | Synchronous sessions |
| Tasks | Asynchronous tasks |
| Connectivity | Connectivity to Second Life |
