# Supplementary material for: Short-term knowledge gains and learner satisfaction after a basic radiology course for family medicine residents in a 3-dimensional virtual environment: a multi-cohort pretest–posttest study in Spain
Source: J Educ Eval Health Prof. 2026 Aug 7;23:25. doi: 10.3352/jeehp.2026.23.25 (PMC13575735; doi:10.3352/jeehp.2026.23.25)
Supplement: Supplementary file 4 [file jeehp-23-25-suppl4.docx]

**Supplement 4.** Results of the multiple-choice tests conducted at the beginning and at the end of the course

| Edition | Paired N | Pre-intervention test | Post-intervention test | Mean change | 95% CI | P-value |
| --- | --- | --- | --- | --- | --- | --- |
| First | 27 | 48.9±10.5 | 59.6±13.8 | 10.7 | 4.0–17.5 | 0.003 |
| Second | 22 | 51.8±10.8 | 57.0±9.6 | 5.2 | 1.3–9.1 | 0.011 |
| Third | 16 | 38.8±10.2 | 57.5±11.8 | 18.8 | 8.1-29.4 | 0.002 |
| All | 65 | 47.4±11.6 | 58.2±11.9 | 10.8 | 6.8-14.9 | <0.001 |

Values are presented as mean±standard deviation and the test results expressed as percentages. Mean change represents the mean within-participant difference between post- and pre-intervention scores. (N) represents the number of participants. P-value represents the probability of error from the Student t-test comparing pre- and post-intervention tests.

CI, confidence interval.
