# Supplementary material for: Short-term knowledge gains and learner satisfaction after a basic radiology course for family medicine residents in a 3-dimensional virtual environment: a multi-cohort pretest–posttest study in Spain
Source: J Educ Eval Health Prof. 2026 Aug 7;23:25. doi: 10.3352/jeehp.2026.23.25 (PMC13575735; doi:10.3352/jeehp.2026.23.25)
Supplement: Supplementary file 5 [file jeehp-23-25-suppl5.docx]

**Supplement 5.** Comparison of baseline characteristics between participants with complete paired assessments and those with pre-course assessment only

| Characteristic | Paired (n=65) | Pre-only (n=15) | P-value |
| --- | --- | --- | --- |
| Pre-course score | 47.4±11.6 | 45.0±11.6 | 0.482 |
| Female | 56 (86.2) | 14 (93.3) | 0.745 |
| Male | 9 (13.8) | 1 (6.7) |  |
| R1 | 34 (52.3) | 6 (40.0) | 0.556 |
| R2 | 19 (29.2) | 4 (26.7) |  |
| R3 | 8 (12.3) | 4 (26.7) |  |
| R4 | 4 (6.2) | 1 (6.7) |  |

Values are presented as mean±standard deviation or number (%). P-values were calculated using the independent Student t-test for baseline knowledge scores and the chi-square test for categorical variables.
