# Supplementary material for: Short-term knowledge gains and learner satisfaction after a basic radiology course for family medicine residents in a 3-dimensional virtual environment: a multi-cohort pretest–posttest study in Spain
Source: J Educ Eval Health Prof. 2026 Aug 7;23:25. doi: 10.3352/jeehp.2026.23.25 (PMC13575735; doi:10.3352/jeehp.2026.23.25)
Supplement: Supplementary file 6 [file jeehp-23-25-suppl6.docx]

**Supplement 6.** List of open-ended comments included in the survey translated into English

**First Edition**

E1-01	Without a doubt, a very creative, useful, and comfortable activity. I would like to have courses like this more often. What I would comment on is the duration. In my opinion, there are too many consecutive hours, during which you inevitably lose focus at times, especially coming from on-call shifts and being tired. My suggestion would be to divide the course into six days instead of three—two for chest X-rays, two for abdomen, and two for musculoskeletal imaging—so each part can be better utilized without losing detail. Once again, thank you for this great initiative and excellent course. Kind regards.

E1-04	I found the course engaging, practical, and useful for my professional development, and I would take it again. I particularly appreciated the sessions focused on clinical cases (chest radiology) and would recommend that future editions place greater emphasis on this approach throughout. On the other hand, in some areas of the classroom, visibility of the main screen content was limited, and it would be helpful to find a way for the displayed content to appear enlarged on our own screens, regardless of avatar position. Overall, I am very satisfied with the course and would benefit from periodically reviewing clinical cases with radiographs.

E1-09	It has been a completely new experience, although I struggle with this type of computer-based format. However, the course overall was very good and I would recommend it.

E1-13	In my opinion, it has been a very interesting and useful course for application in daily practice. Congratulations—it was very engaging and productive. Thank you for what you have taught me and for the method used, which was new to me and more dynamic than those we usually use. Best regards.

E1-14	What I would change about the course is its duration. I think it would be more interesting to spread it over more days with shorter sessions. At times, toward the end of the day, it was difficult to maintain the same level of attention.

E1-15	The chest radiology part seemed the most interesting and useful to me. In the other two sections, I would have preferred a more participatory approach and a focus more aligned with primary care and emergency settings, considering our starting level.

E1-16	Overall, the course seemed excellent, enriching, and essential for our professional practice, especially in emergency care. Perhaps the abdominal CT topics were too in-depth for our level. The musculoskeletal section could have been more participatory; the explanations were brilliant but somewhat dense.

E1-17	As a suggestion, I would include a short introductory session on the first day to learn how to use the platform, as it was somewhat difficult at the beginning. Integrating this into the course program would prevent delays like those experienced on the first day.

E1-18	In my opinion, the chest instructor was the best one. She is excellent at engaging participants and keeping them active throughout the session. Thank you very much for everything!

E1-19	This was my first time using the Second Life platform. I found it to be a very useful tool, as it allowed people from different locations to attend without traveling. The course organization was excellent, and you managed to make it highly participatory despite the distance. Congratulations on your work, and I encourage you to continue offering more editions!

E1-20	In conclusion, it was a very enjoyable and educational experience. I would love to see more activities using Second Life, and even that the annual basic radiology conference could one day be held on this platform. Thank you for everything—you have been excellent instructors.

E1-23	There were difficulties in viewing the board depending on seating position (avatars blocking the view, difficulty adjusting perspective while using the chat, etc.). The correction of assignments was not provided (to our knowledge), and this would have been useful. The best part was chest radiology; applying a similar approach to abdomen and musculoskeletal sections would make them more beneficial. Thank you!

E1-24	I hope a second course will be organized! I feel a strong need for further training in radiology and would be eager to continue.

E1-27	I found the course very interesting, with very useful images and excellent feedback. It might be better to spread the course out over time to allow better completion of assignments and review of the material. The oral presentations were very good overall. I hope more courses like this are offered, including review of covered content and deeper exploration of more complex topics. Very satisfied—I would recommend it to colleagues.

E1-28	From my perspective, I noticed that I was losing attention during the last lecture each day due to the continuous amount of information. In my opinion, it would be better to have sessions of 2 to 2.5 hours every two weeks to better balance with clinical duties. Having access to recorded sessions was very helpful. The practical application of abdominal imaging in on-call settings was particularly useful. Musculoskeletal training is especially relevant given its frequency in primary care and emergency settings.

E1-31	I would suggest not concentrating so many hours of lectures in a single day (although I understand this may be difficult organizationally), or at least including a short break midway through the session. The content was very interesting and dense, and inevitably attention decreased by the end of the day due to fatigue.

E1-34	Overall, I really enjoyed the course, its organization, and delivery. I am a bit disappointed with my results, but expanding knowledge also brings more diagnostic uncertainty. Some questions in the final test had not been covered during the sessions. I would have liked to perform better, but I realize I need to review more. In the trauma section, I would include more than fractures, as these are not the most common cases in primary care (although very relevant for emergency settings). I understand time constraints. These are just suggestions in case there is a follow-up course for those who completed the first one. Thank you very much for your effort and congratulations!

**Second Edition**

E2-01	Overall, I really liked the course. I found it very useful. The only negative point was the chaos on the first day and the connection problems, although these were resolved well on the following days.

E2-03	I found it to be a very good course, as well as the instructors. Perhaps I would have liked more hours of teaching. I am eagerly awaiting feedback on the assignments.

E2-05	I think it would be useful to have supporting materials and follow-up summaries to help with revision. Likewise, live sessions could be recorded and uploaded for later review or for those unable to attend.

E2-08	Creating the avatar was difficult for me, although it was not the most important part. What could be improved is the format of this final questionnaire, as I was unable to complete it until I could open the email on a computer and had time to fill it out on my phone. It is also inconvenient not to receive the course certificate for not completing this survey. I would suggest a format like Doodle or something similar. Otherwise, everything was great. Thank you.

E2-12	What I liked most were Chest’ sessions, which were based on clinical cases and were highly participatory, explaining pathologies and radiographs along the way. Lecture-style monologues become tiring, especially at home where it is easy to get distracted.

E2-14	Due to personal reasons, I was only able to attend the first Wednesday session and complete tasks 1, 2, and 3. In addition, my internet connection did not allow me to access the virtual island properly. I found the format interesting and innovative, but there were some connection difficulties, especially at the beginning.

E2-18	I believe it is necessary to include breaks between presentations in order to maintain a good level of concentration.

E2-22	I really enjoyed the experience. The only drawback was that the sessions felt long, making it difficult to follow the final explanations.

E2-23	I was not familiar with the Second Life platform, but it felt like attending a class without needing a physical location. The main issue I encountered was that I missed the first presentations because my computer was not compatible with the program, although I later used Firestorm without problems after receiving quick support. I particularly liked the sessions where images were shown and participants were asked interactively whether they were normal or not, along with explanations—this encouraged active participation. I also appreciated presentations such as those of musculoskeletal, where he used colors to highlight key lines to focus on, including in chest X-rays. I believe the cost is very affordable, even for residents, and it allows access to training without relying on external sponsorship. Thank you for your work; I would consider enrolling again in another edition to reinforce concepts and images.

E2-24	Overall, the course was quite useful and interesting. The possibility of attending live courses from home was a very satisfying experience that I would definitely repeat. However, I think the assignments should have been downloadable as PDFs, as zooming in on images was difficult, and during the limited free time I had in September, I experienced several connectivity issues with Second Life.

E2-25	The assignments uploaded in the virtual environment were not always visible and could not be viewed if more than one avatar was in front of them.

E2-26	I found the course quite interesting and the format innovative. However, having to move around the virtual building (especially to find assignments or PPTs) was cumbersome and impractical, and it took time to learn how to handle the avatar properly. The sessions were useful, and I learned a lot, especially in chest X-rays. The musculoskeletal section, however, seemed less useful to me. Regarding the abdomen sessions, while the indications for imaging in emergencies were useful, too many CT and ultrasound images were shown, which were less relevant for us, as we usually read reports rather than interpret images directly. In my opinion, it would have been more beneficial to review and interpret more practical abdominal cases instead of so many CT images. Finally, due to my personal situation and on-call workload, completing the assignments at home was literally impossible due to the limited time provided. I believe that allowing a couple of weeks per assignment would have made it feasible and more beneficial.

E2-28	I found the course to be a very good experience; I had never heard of Second Life before. I was able to attend the course even while on vacation in another country. Thank you very much for the initiative, and I hope to participate in future courses.

E2-31	It would be beneficial to offer an in-person ultrasound course, as it is a widely used tool in primary care and emergency settings. It would be very useful to start training early to become familiar with it.

E2-34	I found the course very interesting, with outstanding organization. As a suggestion, I would recommend that most sessions be based on clinical cases (as was done in some sessions). Thank you very much—see you soon.

**Third Edition**

E3-03	My only complaint would be the poor coordination among participants, as we had to stop frequently because someone had technical issues or could not see properly, and especially because slides were being changed while they were being presented. I understand this is not your fault, but it became quite tiring. Otherwise, it was an excellent and very positive experience. Thank you very much!

E3-05	I would have liked the downloadable presentation materials to include more written information, specifying what each image shows or a brief summary of the key points to look for in each case. I am not sure if this is still possible, as it was mentioned that updated PDFs might be uploaded. Overall, I found the activity very interesting and highly useful for my clinical practice. This virtual system is very convenient, although I did experience access issues after updating my computer software. Thank you very much for everything. I would be interested in participating in future activities.

E3-07	Overall, I am very satisfied with the course and found it very useful, although I would have preferred more emphasis on chest and musculoskeletal X-rays, as these are the most relevant and frequent in my professional context in primary care. As a suggestion for future editions, I would recommend shorter daily sessions, even if that means extending the course over more days. Overall, an excellent platform and a very original idea. I will be attentive to future editions.

E3-08	I found the activity very interesting, although I believe the application is not very intuitive. As a suggestion, it would have been helpful if clinical case slides included the corresponding answers immediately afterward, so that when downloading them for review, it would be easier to study.

E3-12	A limitation of Second Life is that if participants do not fully understand how the application works, they may change slides continuously or leave microphones open, making it quite tedious to follow the session and complete evaluations under those conditions.

E3-13	In my opinion, the most useful section was the chest module, as it was much more practical than the others and placed strong emphasis on participant involvement, making the course very engaging.

E3-16	The first assignments seemed difficult and perhaps less useful due to the lack of supporting information, as they consisted only of images. I also hope to receive corrections for the assignments to make them more valuable. Both in chest and musculoskeletal radiology, further depth could be beneficial, although perhaps in shorter sessions (rather than 3 hours), as attention tends to decline.

E3-22	As I always try to provide constructive feedback on my learning experience, here are a few aspects I would suggest improving: Assignments: Participants in the course came from different levels and stages of training. As a first-year resident, I spent a considerable amount of time on the tasks, and at times found them somewhat frustrating—especially during the first week—because, given my limited experience, I was often unsure whether my interpretations of the radiographs were correct or why. One of the strengths of the course is the immediate feedback provided during live sessions and via chat. I would suggest either making the tasks more dynamic (as was done with the evaluations), with time limits and feedback afterward, or designing them as interactive exercises that provide brief explanations when answers are incorrect. I would also recommend recording the sessions for those who are unable to attend live (at least for a few days). Strengths: The course significantly increased participation and concentration by using a new platform and a learning approach that was unfamiliar to us, especially with the opportunity for real-time questions and answers. Overall, I found participants to be more engaged and less afraid of making mistakes compared to traditional classes—possibly due to the use of anonymous avatars. I would like to emphasize how much I enjoyed the entire course, particularly the chest radiology section. The instructor’s teaching approach focused on key concepts, with high and continuous participation, and repeated reinforcement of essential points that we should never forget. I would like to congratulate her, as well as all of you, for forming such a great team and for creating such an accessible and valuable course for residents. P.S. If there is any opportunity to do a rotation with your team at the hospital, even for those of us from other cities, I would be very interested—please let me know! You are excellent educators—thank you very much for everything!
