# Supplementary material for: Short-term knowledge gains and learner satisfaction after a basic radiology course for family medicine residents in a 3-dimensional virtual environment: a multi-cohort pretest–posttest study in Spain
Source: J Educ Eval Health Prof. 2026 Aug 7;23:25. doi: 10.3352/jeehp.2026.23.25 (PMC13575735; doi:10.3352/jeehp.2026.23.25)
Supplement: Supplementary file 7 [file jeehp-23-25-suppl7.docx]

**Supplement 7.** Key subcodes for first-level codes (advantages, disadvantages, and suggestions) in open-ended residents feedback

| **Subcode** | **Explanation** | N |
| --- | --- | --- |
| **Advantages** |  |  |
| Usefulness and practical relevance | The course was perceived as useful, engaging, and enriching, with clear applicability to daily clinical practice, particularly in emergency care settings. | 19 |
| Platform benefits and accessibility | The virtual format was perceived as innovative, convenient, and accessible, enabling remote participation and offering a cost-effective alternative to traditional training. | 7 |
| Chest X-ray module relevance | The chest radiology module was identified as the most useful, practical, or engaging content area | 7 |
| Teaching methodology | The case-based, dynamic, and interactive format of the sessions was positively valued by participants. | 5 |
| Teaching quality and instructor performance | The instructors were highly valued for their clarity, ability to engage learners, and the quality of feedback provided. | 4 |
| Learner engagement | The format was perceived as stimulating participation, concentration, or active involvement | 2 |
| **Disadvantages** |  |  |
| Platform technical/usability problems | Initial technical problems, connectivity issues, incompatibility, or poor intuitiveness of Second Life | 8 |
| Session length and cognitive fatigue | Sessions were perceived as too long or too dense, leading to loss of attention or tiredness | 6 |
| Interaction issues | Problems caused by other participants or the virtual setting, such as slide changes, open microphones, or blocked view | 3 |
| Feedback issues | Assignments lacked support information, correction, or enough orientation to be useful | 3 |
| Content issues | Some topics were considered too advanced or less relevant, especially abdominal CT/ultrasound or some MSK content | 3 |
| Administrative issues | Problems related to administrative aspects, such as certification requirements or survey completion. | 2 |
| Task burden | Assignments were difficult to fit around workload/on-call duties or required too much time | 2 |
| Navigation barriers | Difficulty navigating the virtual environment and locating tasks or materials. | 1 |
| **Suggestions** |  |  |
| Schedule redesign | Redistribution of the course schedule into more days with shorter sessions. | 6 |
| Content adjustment | Increase focus on chest, MSK, and ultrasonography topics, and better adapt content to primary care and emergency practice | 5 |
| Support materials | Provide summaries, annotated slides, written explanations, or answers to cases | 4 |
| Methodology improvement | Increase participatory, case-based teaching, especially in Abdomen and MSK | 4 |
| Recorded sessions | Make live sessions available as recordings for later review | 3 |
| Include breaks | Inclusion of breaks during sessions | 2 |
| Platform training | Initial training on how to use the platform | 1 |
| Task redesign | Improve assignments through correction, immediate feedback, or more interactive design | 1 |
| More time for tasks | Extend deadlines or allow more time to complete tasks | 1 |

CT, computed tomography. MSK, musculoskeletal.
